# The Natural Environmental Factors Influencing the Spatial Distribution of Marathon Event: A Case Study from China

**DOI:** 10.3390/ijerph17072238

**Published:** 2020-03-26

**Authors:** Zhanbing Ren, Yifan Zuo, Yudan Ma, Mu Zhang, Lee Smith, Lin Yang, Paul D. Loprinzi, Qian Yu, Liye Zou

**Affiliations:** 1 Department of Physical Education, Shenzhen University, Shenzhen 518061, China; rzb@szu.edu.cn; 2 School of Management, Jinan University, Jinan Guangzhou 510632, China; yifanzuo@stu2019.jnu.edu.cn; 3 Shenzhen Tourism College, Jinan University, Shenzhen 518053, China; zhangmu@jnu.edu.cn; 4 Exercise Science Research Center, Jilin Institute of Sport Science, Changchun 130022, China; yudanma@sina.cn; 5 Cambridge Centre for Sport and Exercise Sciences, Anglia Ruskin University, Cambridge CB1 1PT, UK; Lee.Smith@anglia.ac.uk; 6 Cancer Epidemiology and Prevention Research, Alberta Health Services, Calgary, AB T2S 3C3, Canada; Lin.Yang@albertahealthservices.ca; 7 Department of Health, Exercise Science and Recreation Management, The University of Mississippi, Oxford, MS 38655, USA; pdloprin@olemiss.edu; 8 School of Psychology, Shenzhen University, Shenzhen 518061, China; yuqianmiss@163.com

**Keywords:** marathon running, natural environmental factors, coupling analysis, geographic information systems

## Abstract

*Purpose:* The purpose of this study was to investigate the influence of natural environmental factors on the spatial distribution of marathon events in China, and to identify the suitable natural environmental factors for the marathon events. *Methods:* Geographic information system (GIS) spatial analysis tools were used to perform coupling analysis, e.g. overlap, neighborhood, intersection and buffer for terrain, climate, air quality, mountains and water resources with 342 marathon events held in China in 2018. *Results:* The results indicate that the spatial distribution of marathon events in China is negatively correlated with the elevation of the terrain (plain > hill > plateau > mountain > basin); climate (subtropical monsoon climate > temperate monsoon climate > temperate continental climate > tropical monsoon climate > plateau alpine climate), air quality (level 3 > level 2 > level 4 > level 1). Results indicate that buffer zones can protect water resources: there are 24 items in the buffer zone of river 0.5 km and lake 1 km, 131 items in the buffer zone of river 3 km and lake 5 km, 191 items in the buffer zone of river 5 km and lake 10 km, 298 items in the buffer zone of river 10 km and lake 20 km. Results indicate for mountain range buffer: 13 items in the 20 km buffer and 39 items in the 50 km buffer. *Conclusions:* Marathon events are more likely to be held on the third rung of China’s topography where a city has a typical landform (plains, basins, hills, or mountain) with good climate and air quality. Meanwhile a city with water and mountain resources for recreational events such as cross-country or obstacle course are essential. The contribution of this study is to systematically and intuitively reflect the influence of natural environment factors on the distribution of marathon events in China, and to provide evidence for the medium and long-term planning of marathon events in China, the selection of venues for different types of marathon events and how to attract participants.

## 1. Introduction

As an outdoor event, marathon running is influenced by natural environmental elements. Natural environmental elements are non-artificial basic material components in nature that directly or indirectly impact the human life and production environment, which are independent, differ in properties, and have a general pattern of evolution. The common natural environments include water, atmosphere, sunlight, soils, and mountains [1]. The research subjects of natural environments mainly include the climate [2], weather [3], water resources [4], animals and plants [5], agroforestry [6], geomorphology [7], and landforms [8]. Researchers have explored the relationship between the natural environment and marathon running from different perspectives, such as the feasibility of marathon running in different natural environments, Chang et al. found that the marathon can be held in combination with local environmental advantages. The natural environment of Yan’an has obvious advantages, and the venue can be integrated into the natural landscape [9]; marathon runners’ motivation and cognition in natural environments, Peric et al. selected the Fruska Gora marathon as the research object, and found that the major factors influencing the self-experience of marathon included the convenience of the activity tourism destination, the contribution of the activity to tourism development, and the natural environment [10]; Vanos, et al. studied various factors affecting human thermal comfort during marathon by applying the human energy budget model. They found thate air and surface temperature, solar radiation, humidity and wind speed all affected human thermal comfort, among which high humidity and low airflow aggravated human discomfort [11]; the impact of different natural environments on the participants’ physical and mental conditions, Xiaohua and Feng found that training on the plateau not only ensured stability during the race, but also provided a solid psychological foundation [12]; Kosaka et al. Study marathon runners and spectators are faced with the problem of high temperature and pressure, the COMfort FormulA and the Human Heat Balance model to evaluate potential Heat stress on marathon athletes, the results showed that the climate of July and August, sunny games, the course were named as “dangerous” or “extremely dangerous” in the second half of the race, has 10 km of the part that persists for more than “extremely dangerous” level [13]; the impact of marathon running on different natural environments. When studying the risk identification of marathon events, Jun and Zhang identified environmental and natural risks [14]; the impact of different terrains on marathon runners’ performance. The pace selection, the degree of slope and running speed of marathon runners on uphill and downhill sections are significantly correlated to their performances [15]; and altitude training that can improve the physiological functions of marathon runners and enhance their performance [12,16]. Indeed, research has found that marathon events are mostly distributed in spring (week 14 to 17) and autumn (week 41 to 44) and the average temperature of host cities varies close to optimal value for long distance races [17]. Some studies have analyzed the changing trend of meteorological values of marathon such as temperature, humidity, air quality, and wind speed over time, and found that the climate has an impact on participants’ physical sensations and performance in the race [11,13,18,19,20]. There is also a large body of literature investigating the impact of different climates on the physical conditions of marathon runners. Research has found that factors such as the rainfall, humidity, wind speed, temperature, air pressure, and wet bulb globe Temperature index during the marathon running are correlated to sudden cardiac deaths [21]. Research in relation to water resources and mountains in the host cities, has mainly focused on the influences of these environments on hosting marathon events, specifically how to attract more participants. For example, it has been suggested that the Boston Marathon attracts participants owing to the New England hilly terrain [22]. 

At present, most of the studies on marathon running and the natural environment in China are from the perspective of urban development and on the micro level, such as the impact of the development of urban marathon running on the natural environment [23,24,25,26,27]. Chinese scholars have found that marathon running in China are mainly distributed in plains or mountainous regions (except Lhasa), where the climate and air quality index are high-level [28]; the climate and natural conditions in Tibetan Plateau is not ideal for holding marathon running; the number of marathon runners and events decreases from the eastern coast to the western inland area, and the natural environmental element is the main influencing factor. It is also concluded that other factors influencing the spatial distribution of marathon events included economic strength, tourism development level, social development level and population base [29].

In terms of spatial data processing, geographic information systems (GIS) represent an effective spatial analysis technology. It can store, manage, and retrieve spatial data and establish a spatial database. The database includes a spatial database and a spatiotemporal database GIS can realize comprehensive analysis and data processing to acquire useful information for spatial analysis. Meanwhile, it can also visualize the result. To date, there are few studies utilizing GIS to explain the impact of natural environmental elements on the distribution of marathon events in China. When considering the growing popularity of marathon events in China and the increase in participation it is important for sustainability to understand which natural environmental parameters are associated with marathon event location choice. The aims of the present study were to: (a) use GIS-related spatial analysis tools to perform coupling analysis on marathon running in China which are recognized by the Chinese Athletic Association (CAA) from the perspective of terrain and topography, climate and air quality, and mountains and water resources in China; (b) explain the potential influencing reasons of natural environmental elements on the spatial distribution of marathon events in China; (c) discuss, from a public health perspective, why such natural environments are suitable for marathon events based on the underlying causes.

## 2. Materials and Methods

### 2.1. Data Sources

In 2018, the total number of marathon events held in China was 342 and the total number of participants was approximately 3,362,590 (http://www.runchina.org.cn/) [30]. These data do not separate, professional and the amateur marathon events. Owing to weak data reporting relating to amateur events, the participants of nine marathon events, including 2018 Yan’an International Courtyard Marathon and 2018 Shaoxing Shangyu Caoejiang International Half Marathon, were missing. Marathon events by month, participants and the total events in each province are reported in Table 1. 

### 2.2. Research Methods

Spatial analysis is a commonly used analytic approach in geographic information science. Spatial analysis is often used in relevant social studies as it can provide accurate cognition, evaluation, and comprehensive understanding of the interaction between spatial location and space [31]. The comprehensively calculates spatial data using attribute information, and finally extracts spatial information. The aim is to reveal the features and commonalities of data through visualization techniques and it is usually used to identify abnormal points or areas and it detects the pattern, aggregation, and hot spots of spatial data [32]. ArcGIS10.3 (Esri, Redlands, CA, USA) was used in the present study to visualize the spatial geographic distribution of marathon events hosted in China in 2018 and the map is detailed to provincial administrative regions. First, for accuracy, the marathon running site information were collected and the basic information were matched on Baidu Map (a web mapping service similar to google map, provided by Baidu, China); after confirmation of an accurate match, the addresses can be deemed accurate to county level. Meanwhile, to ensure the accuracy and the authority, the base maps used in this study are all from the Resource and Environment Data Platform of the Chinese Academy of Sciences, which is devoted to providing help to studies on the resource and environment research in China and helps to effectively process and integrate relevant spatial information data.

#### 2.2.1. Overlay Analysis

When carrying out overlay analysis, in a unified spatial coordinate system, new data is generated through performing set operations on two or more sets of data. The aim of overlay analysis is to analyze the interrelationship between the spatial features and attributes of spatial objects which are correlated in terms of spatial location. Specifically, to overlay the data on the same map to generate a form of spatial relationship and an attribute feature relationship, thus to discover the difference, relations, and changes of the multi-layer data [33]. There are five types of overlay analysis: visual information overlay, point-polygon overlay, line-polygon overlay, polygon overlay, and raster layer overlay. Intersect is used in ArcGIS10.3, which is a tool for calculating the geometric intersection of input features. Features or portions of features which overlap in all layers and/or feature classes will be written to the output feature class [33,34,35]. This research adopts overlay analysis to study the interaction and coupling relationship between marathon running and the natural environmental elements. 

#### 2.2.2. Proximity Analysis

This study uses the Euclidean distance method, which calculates the linear distance between two points on the Cartesian plane by measuring the distance in the two-dimensional Cartesian plane. As the size of the buffer depends on the radius R, the buffer area of Oi object can be defined as [36]:

Bi={x:d (x, Oi) ≤ R}(1)

Namely the radius of R object Oi Buffer area is all the distance d from Oi less than or equal to the point of R collection, d refers to the minimum Euclidean distance generally [36]:

O = {Oi: i = 1, 2, 3,...., n}(2)

The radius of the buffer zone for R is a combination of single object of the buffer zone, namely [36]:

(3) $$B=\overset{n}{\underset{i=1}{⋃}}Bi$$

This study establishes buffers for water and mountain resources and calculates the number of marathon running within the distance areas.

## 3. Results

### 3.1. Terrains/Landforms and Marathon Running

**Figure 1:**
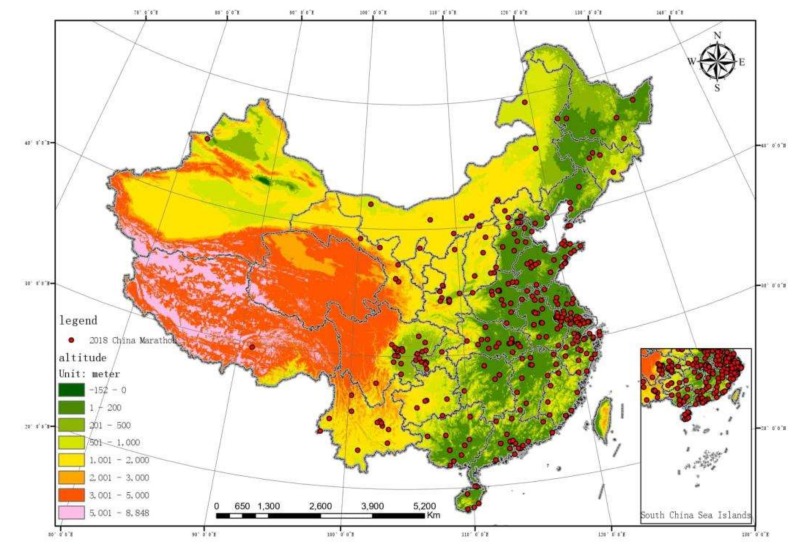
Terrains and marathon running coupling diagram.

**Figure 2:**
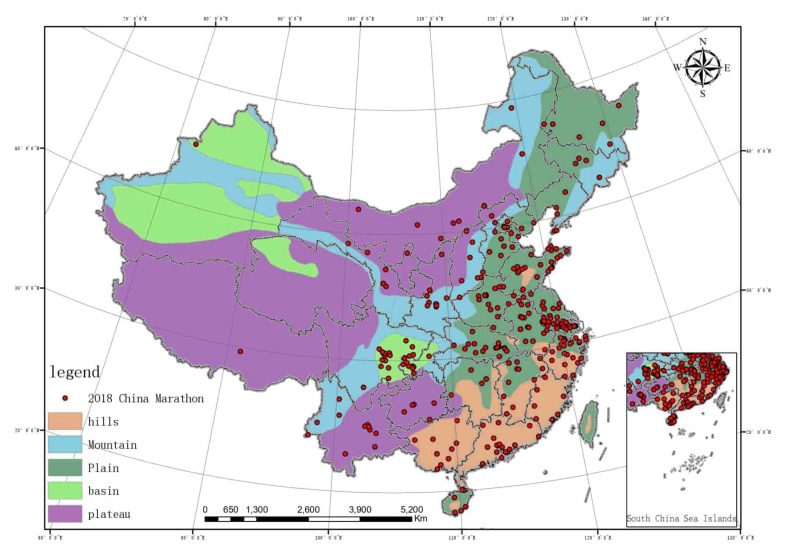
Landforms and marathon running coupling diagram.

The spatial distribution data of the altitude of China (DEM) was acquired from the geospatial data cloud (http://www.gscloud.cn/) [37] and the vector map of China’s terrain was created using ArcGIS10.3. The red, yellow, and green gradient colors indicate different altitude. The features of marathon events in China were also imported (Figure 1). The China Topographic Map in the preface of China Topographic 3D was used as the image registration. Five topographic features are shown in different colors: hills (light brown), mountains (blue), plains (dark green), plateaus (purple), and basins (light green). The marathon running features of China were imported (Figure 2).

As presented in Figure 1, the highest frequency in marathon events are in the green area, followed by the yellow area and the red one. Therefore, the number of marathon running is the largest in the third terrain step and the smallest in the first. This indicates that the spatial distribution of the marathon event in China has a significant negative correlation with the altitude. 

Fewer marathon events were observed at high altitudes when compared to low. It can be clearly seen from Figure 2 that a higher frequency of marathon events are hosted on plains than on other landforms, with plains > hills > plateaus > mountains > basins (Table 2).

### 3.2. Climate/Air Quality and Marathon Events

**Figure 3:**
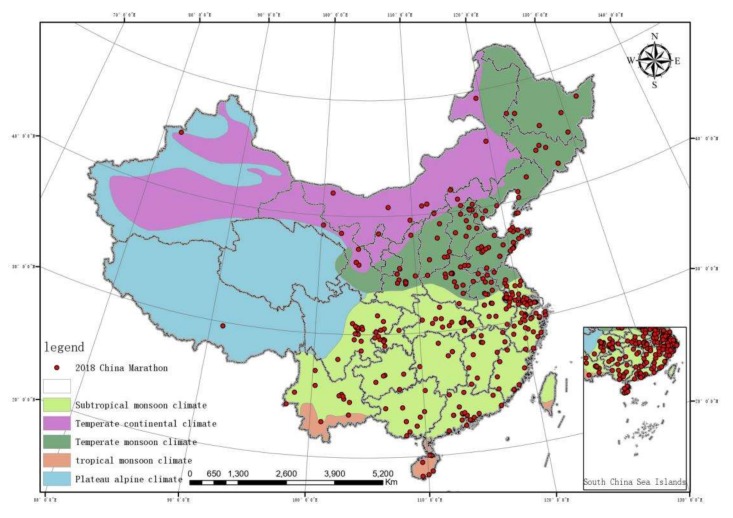
Climate and marathon running coupling diagram.

China’s climate classification map was produced based on the features of the climate in China and the China’s climate zoning data launched by the Chinese Academy of Sciences ’Resource and Environmental Science Data Center. Different colors indicate different climates, which are tropical monsoon climate (light brown), plateau alpine climate (blue), temperate monsoon climate (dark green), temperate continental climate (purple), and subtropical monsoon climate (light green). The vector map was generated and the features of marathon running were imported to generate Figure 3.

From 1 January to 9 November through ArcGIS10.3, the comprehensive meteorological indexes of 31 provinces and direct-controlled municipalities were divided into four meteorological tiers from good to bad by the means of Jenks (The specific division criteria are shown in Table 3.). 

**Figure 4:**
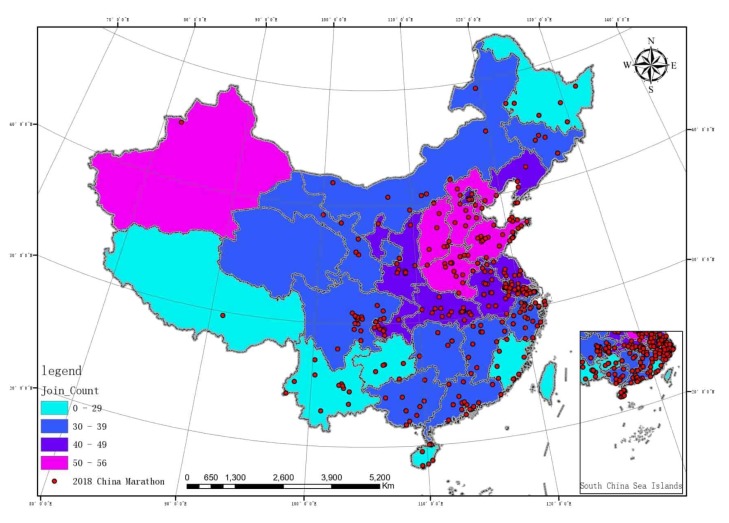
Air quality and marathon running coupling diagram.

The result is based on the data of 376 cities and 1499 monitoring points provided by China Weather (http://www.weather.com.cn/) China environment monitoring station [38]. The station includes 35 monitoring points in Beijing Environmental Protection Monitoring Center, 28 monitoring points on Tianjin environment and air quality GIS launching platform, 197 monitoring points in Hebei air quality auto monitoring and launching system, 166 monitoring points on Zhejiang environment and air quality index launching platform, and 182 monitoring points on Shandong city environment and air quality index information launching platform. First-tier areas have the best air quality with a value of 0–29; fourth-tier area has the worst air quality with a value of 50–56. The features of marathon running in China were imported and Figure 4 was generated.

In the climate and marathon event coupling diagram, it can be seen that marathon running in China are mainly held in monsoon climatic areas, mostly temperate monsoon climatic and subtropical monsoon climatic areas. Subtropical monsoon climate > temperate monsoon climate > temperate continental climate > tropical monsoon climate > plateau alpine climate. Please see the details in Table 3. In the air quality and marathon event coupling diagram, the host cities are mainly distributed in second-tier (30–39) and third-tier (40–49) areas. Third tier > second tier > fourth tier > first tier. Please see the details in Table 3. Meanwhile, the air quality index of each province was determined as the independent variable, and the number of marathon events held in each province as the dependent variable, and the regression analysis was conducted. The results are: R = 0.216, R^2^ = 0.047, indicating that the fit of the regression model is not good. The significance of variance analysis was 0.243, indicating that there was no significant linear relationship between air quality index and the number of organizations in this analysis. The significance level in the t test is 0.601, indicating that the coefficient of this regression equation is not significant and not statistically significant.

### 3.3. Water Resources/Mountains and Marathon Running

**Figure 5:**
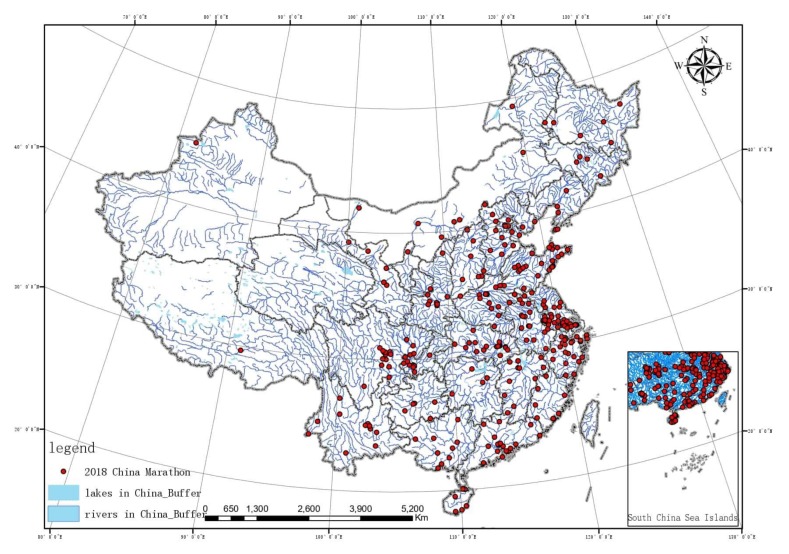
Water resources and marathon running coupling diagram.

**Figure 6:**
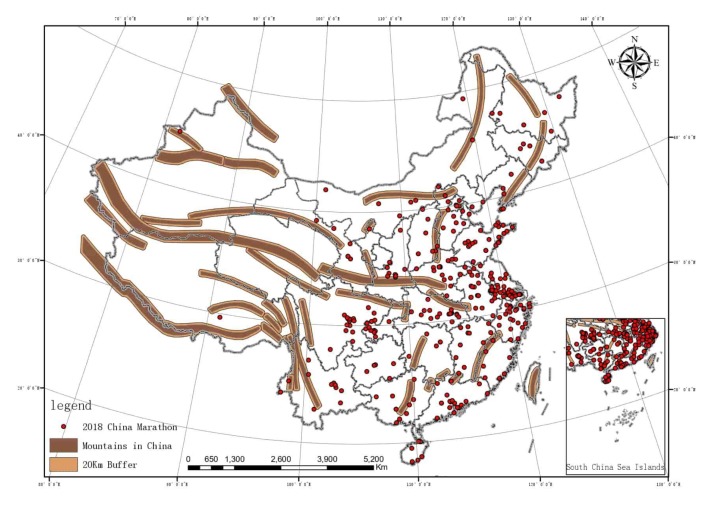
Mountain ranges and marathon running coupling diagram.

To observe the hydrological distribution of China more intuitively, according to the River Basin Spatial Distribution Dataset of China Extracted on the Basis of DEM published by Chinese Academy of Sciences Resource and Environment Science Data Center, the main rivers in China were extracted in ArcGIS10.3 and a buffer area with a radius of 500 m was established. The major lakes were extracted to establish a buffer radius of 1000 m. Marathon running features of China were imported to generate Figure 5. In order to explore the relations between the spatial distribution of large mountain ranges in China and marathon running, the geographical coordinates of major large mountain ranges in China were collected to generate planar features. In ArcGIS10.3, the buffer area of the mountain range was adjusted to 20 km and the features of marathon running in China were imported to generate Figure 6.

As shown in Figure 5 and Figure 6, the overlapping results were obtained through the intersect analysis of the buffer area features and marathon events: (1) there are 24 marathon events intersecting with the water resource buffer area and 13 marathon events intersecting with the mountain buffer area. In terms of the attribute of the events, among the 24 marathon events intersecting with water resource buffer area, 18 are distributed in the exoreic river area, nine marathon events were related to water, Example: Chongqing Dazu runs around Longshui Lake half marathon Chongqing Dazu Ring Longshui Lake Half Marathon. 19 are held in the cities, and 5 are held in tourism sites.

Among the 13 marathon events intersecting with the mountain buffer area, 9 are mountain marathon events, but there are still four city events, which are held in Dali, Xi’an, Yinchuan, and Huolingol respectively. It is shown in the output result that neighboring coefficients of marathon events and water resources and mountains are not high, Intersect with the two buffers only account for 7.0% and 3.8% of the total, respectively. Therefore, the radius of buffer area was extended, with the radius of rivers being extended to 3 km, 5 km, and 10 km, lakes to 5 km, 10 km, and 20 km, and mountians to 50 km. Intersect analysis was performed again and the output is shown in Table 4.

Then, the buffer distance of rivers, lakes and mountains was determined as the independent variable, and the number of marathon RACES held at each distance was determined as the dependent variable. Regression analysis was carried out respectively. The results are shown in Table 5:

The R and R^2^ of the three results are above 90%, indicating that the fitting effect of the regression model is very good. The t-test result *p* < 0.05 for the distance between the buffer zone of the mountain range was very significant, while the t-test result *p* < 0.1 for the river buffer zone and lake buffer zone was not significant.

## 4. Discussion

### 4.1. Correlations Between Terrain/Landforms and Marathon Events

Generally, China’s terrain is staircase-shaped, with the west being higher and the east lower, extending to the east from Qinghai-Tibet Plateau to the continental shelf below the sea surface, which makes it easier for the humid ocean air to go into the land and form rainfall. The terrain in China can be divided into three steps: the first step runs across the Qinghai-Tibet plateau in southwestern China, with an average altitude above 4000 m; the second step covers Kunlun Mountain and Qilian Mountain in the north and Hengduan Mountain in the east, with an average altitude of 1000–2000 m and under 500 m in some area, so the terrain declines significantly; the third step covers the area to the east of the second step, including from the Daxinganling Mountain, Taihang Mountain, Wu Mountain, Xuefeng Mountain to the continental shelf, with an average altitude under 500–1000 m [39]. Having a vast land, China has diversified and complex terrains and all five landforms including plains, plateaus, mountains, hills, and basins. These various landforms are scattered and intersected with mountains being the skeleton. It can be seen from the topographical results that the marathon event in China are mainly distributed on the third terrain step, indicating that the altitude has an impact on marathon running. The low-lying areas are more suitable for the performance and spread of sports activities. The pattern is to radiate outward from a culturally developed area. It is possible that it is problematic to host marathon events in areas with higher altitude due to the monotony of culture, conservative social ideas, and economic status. Mostly, the marathon events held on the third step emphasize local features, such as the Lhasa Half International Marathon, the Linze Ecological Marathon, and the Lanzhou Lily Road 100 km Ultra-Trail. At the same time, the study found that the quality of supporting facilities, transportation quality, track cleanliness, media publicity and other indicators can affect the participation motivation of marathon [10]. In addition, third tier regions are more developed and thus more suitable for holding marathon events than those first and second regions. The economy is relatively developed among the third tier regions with sufficient allocation of public resources including public health facilities, public sports facilities, public cultural facilities, public transportation facilities, public green facilities (specifically urban roads, Bridges, city square, city lights, road signs nameplate, urban greening, urban scenic spot, urban park). The altitude of the terrain was inversely proportional to the number of marathon events held. The Sichuan Basin can also support this assumption. Although the Sichuan Basin is located on the second step, due to the low altitude, the openness of ideology and the level of economic development are higher than other areas on the same step.

The results relating to landforms show that most marathon events are held on the plains, which indirectly indicates that plains may be more suitable for holding such events. The gentle terrain can guarantee the performance of the runners. Studies have shown that as the safety of a track increases, runners are able to be more focus on running. However, the environment with high recognizability often has different characteristics, which can bring people a good sense of pleasure, such as the broad riverside avenue and the historic street with strong culture [40]. To analyze from a higher level, in the plains, the high economic level and open location are beneficial to hosting events. Next are hills, plateaus, and mountain areas. People are used to referring to hills, mountains, and rugged plateaus as mountainous areas. From the perspective of marathon event resource development, in recent years, the mountainous area has also become a hot spot. For example, Youzhou 100 Ultra-Trail and Yellow River Shilin 100 Ultra-Trail are both long-distance endurance races, where the participants overcome the difficulties in different natural environments and run in the fastest speed to get a best ranking. This type of events is integrated with the natural environment, but affects the performance of the runners [41]. In mountainous areas, marathon races are in the level, uphill and downhill gradients due to the high ground undulations, so the runners’ speed selection is also different from that of the flat race [15]. Studies have observed mountain super marathon run athlete the influence of the medial meniscus extrusion for people’s health, and the results that mountain super marathon runner (without symptoms of knee joint, knee joint injury or surgery) observed under the limit load of the medial meniscus extrusion is a kind of temporary and reversible phenomenon, with the passage of time is completely reversible [24]. But the concerns of the public is still inevitable, participating in the marathon will cause certain influence to the body, some studied has reported muscle damage and detrimental influence on inflammatory biomarkers and related biomarkers of heart injury in proportional to the running distance up to 72 hours upon competing 54 km and 111 km super endurance mountain races [25]. Therefore, when choosing such terrain events, runners need to think more about their own health conditions, while the organizers need to spend more time on route design and well-equipped medical treatment. As can be seen from Figure 2 and Table 2, Relative to other terrain, marathon events are less distributed in basin terrain. 

### 4.2. Correlations Between Climate/Air Quality and Marathon Events

Having diverse climates, China generally shows an obvious monsoon climate. The prevailing wind in winter and summer are significantly different, and the monsoon precipitation also varies along with the seasons [42]. As can be seen from the result of climate, marathon events in China are mainly distributed in temperate monsoon climate regions and subtropical monsoon climate regions, while less are held in temperate continental climate regions, tropical monsoon climates regions, and plateau alpine climate regions. Monsoon simply refers to seasonal wind, including many factors such as temperature, humidity, rainfall, sunlight, and air pressure. Generally speaking, in monsoon climate regions, the temperature is moderate, the weather is humid, and the hydrological conditions are desired. Such weather makes people relax and encourages people to do some entertaining leisure activities. When the athletes running the marathon, their bodies feel more comfortable [43]. In temperate continental climate and plateau alpine climate regions, the temperature is low, the weather is cold, the rainfall is low, and it’s often windy and sandy. It is unsuitable to hold marathon events in such weather condition. In the tropical monsoon climate regions, the temperature is high, so most runners believe that if it’s not the high temperature, they would have better performance [43]. Studies have indicated that disadvantageous environmental elements such as rainfall and excessive humidity may affect the performance of athletes and increase the possibility of injury when doing sports [44]. High wind speed and low humidity can increase the possibility of asthma [45]. Therefore, during marathon running, the wet bulb globe Temperature index level is shown such as for the New York International Marathon. Since the year 2000 The China Meteorological Administration has recorded weather conditions when marathon events are held in Beijing. Only two events encountered rain and the temperature on the marathon days was optimal (16.8 °C~10.9 °C). In Beijing marathon events, influenced by the meteorological factors, data has shown that the athletes’ running times can vary in a range of 9 to 12 min. Studies have shown that it is best for the athletes to perform optimal when the temperature is between 14 °C and 16 °C, the humidity is between 30% and 60%, the air pressure is between 1015 and 1023 hPa, and the wind speed is between 2 and 5 m/s [43]. This is also likely why events in China are mainly held in April, October, and November. In these three months in most regions, the temperature is usually between 14 °C and 16 °C.

Air quality directly reflects the degree of air pollution. Classified by air quality, nearly 70% of marathon running in China are held in the second tier and third tier. Studies have found that severe outdoor air pollution causes 3.3 million premature deaths worldwide each year [46]. It not only causes direct damage to lung functions [47], but also indirectly affects the cardiovascular system [48] and aids in the development of osteoporosis [49]. Long-term outdoor sports in an environment with poor air quality will likely increase the rate of lung function decline. Although exercise can increase the lung capacity and reduce the arterial stiffness, in an environment with poor air quality, carbon particles and the ultrafine particles in the waste gas of diesel may negate the benefits of exercising [50] and largely increase the risk of having cardiovascular and osteoporotic diseases. Therefore, it is suggested that the air quality should be considered before, during, and after a marathon event. Meanwhile, before hosting a marathon event, it is recommendable that the government plan and improve local air quality out of consideration for participants’ health.

### 4.3. Correlations Between Water Resource/Mountains and Marathon Events

In this study, there are numerous rivers and lakes in China, and the distribution is uneven. The river network density is higher in the south than in the north, and higher in the east than in the west. The runoff in China, besides some glacier melt in the alpine areas, are mostly atmospheric precipitation. The mountains are vain-shaped [51]. The mountain range in China constructs the skeleton of China’s geography, topography, and terrain and usually acts as the boundary of different landforms. The major mountain ranges are distributed in the middle and west part of China and located in the first and the second step, including Altai Mountains, Tian Shan, Kunlun Mountain, Karakorum Mountain, Himalaya, Yin Shan, Qinling, Nanling, Daxinganling, Changbai Mountain, Taihang Mountain, Wuyi Mountian, Taiwan Mountain, and Hengduan Mountain.

Water resources include rivers and lakes. Due to the topography of China, many rivers flow eastward, connecting the east and the west. Water resources and mountain resources are generally selected as venue resources in sports events [52]. For marathon events, although water resources cannot be used as the venue directly, they can serve as views along the running track to attract more participants. From the perspective of the majority’s motivation, runners all want to achieve the purpose of entertainment, recreation, relaxation and forgetting daily affairs, while the combination of natural scenery and marathon track is found to be easier to achieve such purpose [10]. Among the 342 marathon events, only 24 were held by water resources, and only nine of them used the water resource as an attraction. When extending the buffer radius, it was observed that many races can be associated with water resources. Under the condition that the buffer radius of major rivers is 10 km and that of major lakes is 20 km, it is reflected that areas with better hydrological conditions are more likely to become a human settlement. The population is large in these areas, which is a necessary condition for abundant leisure and sports activities. In the history urban development, nature has nurtured rivers, and rivers have nurtured cities. The emergence and prosperity of cities has a significant correlation with nurturance of rivers. Buildings and trees that generate low sky view factors on city roads and sidewalks can protect viewers from radiant heat in the morning [11]. Today, to protect the rivers in urban areas, many cities have set a goal of “clear water, green shores, and smooth flow” (http://www.xiangxiang.gov.cn) [53], which provides more choices for marathon running. For example, Changsha International Marathon event set the track along the Xiang River. Organizers usually take advantage of the unique natural landscapes to carry out sports events with local features. It is a main trend for the sports events, sports tourism, and sports leisure to be combined with the unique local natural and climatic views, such as the karst landforms in Guizhou Province in China [54]. Present findings suggest that most mountains in China are located on the boundary between the second and the third terrain step. Although mountain resources provide a good natural environment for marathon event tourism, only few events are held by the major mountains out of consideration for the competition results. However, as more marathon running events aim to develop the signature of the city, popularize the concept of national fitness, and promote sports tourism, more events are now held in the mountain areas, with the majority being trail running and obstacle races. From the perspective of public health, the marathon track requires a low-lying, gentle track. Firstly, it is beneficial to the health of runners. Secondly, the economy of such areas is relatively developed, the allocation of social public resources is correspondingly sufficient, and public health facilities are relatively complete. The choice of climate and air quality is closely related to the performance and physical health of the participants. The appropriate weather is most conducive to the athletes to exert their physical ability and achieve good performance, while the poor air quality is very damaging to the heart and lungs. When it comes to the choice of mountains and water resources, the combination of natural scenery and marathon track can achieve the purpose of entertainment, recreation, relaxation and forgetting daily affairs. However, in cities, water resources can often be surrounded by the track to connect urban greening, urban scenic spots and urban parks.

Although currently most sports events tend to have a negative impact on natural environment, through the analysis of environment’s impact on marathon events, this study suggests that natural environments are important to the sustainability of sports events. Many participants of sports events held in “nature” argue that small-scale outdoor competitions which “Leave No Trace” will not have an impact on the natural environment, but this assumption remains untested [55]. The impact of climate and air quality on marathon running is also reflecting that the host city and the organizer may take the advantage of holding the race to improve local environment. As the 2022 Winter Olympics is approaching, Beijing encounters a new opportunity to promote its ecological civilization, including the impact of environment protection on climate and air quality [56]. These measures are also beneficial to marathon running in Beijing. Although hosting major sports events may enhance the reputation of the host country and the host city, promote the urban development, stimulate the economy, and improve the infrastructure, the environment may be damaged by human activities due to negligence [57]. Many scholars and government agencies have started to research excessive utilization of the outdoor sports resources and will surpass environmental carrying capacity. estimate the threat to ecological safety after overbearing [58], and evaluate the environmental performance of sports events [59,60]. These issues are also applicable to the studies on marathon events and should be further explored. Currently marathon races held in the rich natural resources and suitable environment is limited. In contrast, due to the commercial value, economic interests, and many other factors, races held in cities with poor air quality is more. As I also mentioned while marathon as a sport, is originally held in China in order to promote the concept of the national fitness, but for a long time in the air quality is poor under the environment of outdoor sports could increase the rate of lung function decline, is not conducive to health. But the Chinese government formulated a series of measures to improve the urban living environment, according to China’s air quality improvement report statistics. In 2013, China will be the Beijing and Tianjin wing, Yangtze river delta and the Pearl River delta region designated as key areas for the control of air pollution, from the national level to carry out the regional atmospheric pollution zone spreading, promote regional air quality to improve year by year. By 2018, the average PM_2.5_ concentration in the three key areas had dropped by 48 percent, 39 percent and 32 percent, respectively, compared with 2013. The average PM_2.5_ concentration of nine cities in the pearl river delta has reached the ambient air quality level ii standard for four consecutive years since 2015. The concentration of PM_2.5_ in Beijing has dropped sharply since 2013, from 89.5 micrograms per cubic meter to 51 micrograms per cubic meter in 2018 [61]. Therefore, the quality of urban air environment is improved, which is more conducive to the holding of urban marathon events, and the conflict between economic value and health value is gradually reduced.

All in all, from the perspective of the development of the natural environment, the Chinese Marathon running can hold more events in provinces or cities with the following characteristics in the future: (a) It is located on the third rung of China’s topography; (b) Belonging to plains, basins, hills, and mountain areas; (c) A city with good climate and air quality; (d) A city with water and mountain resources for recreational events such as cross-country or obstacle course. Meeting condition a indicates that the host area has a complete allocation of social and public resources, and the economic and social benefits brought by the event are also greater. According to condition b, the track is also more identifiable and safer, reducing the occurrence of sports injuries. Meet the condition c, indicating that the performance of the competitors can be better at the same time, the health can also be guaranteed accordingly. d. Perfect combination of natural scenery and marathon course, so as to achieve the experience of entertainment, recreation, relaxation and forgetting daily affairs. The provinces that can meet all the above conditions are Zhejiang, Jiangxi, Guangxi, Heilongjiang and Hunan. Jiangsu, Guangdong, Hubei, Sichuan, Henan, Yunnan, Hebei, Fujian, Shanghai, Hainan and Liaoning met the two criteria. All belong to the provinces suitable for holding marathon events, but the specific host places still need to be considered from other aspects. There are still plenty of marathons in Shandong, Anhui, Beijing, Shaanxi, Chongqing, Gansu, Shanxi and Inner Mongolia. In contrast, the number of marathons held in Heilongjiang, Hunan and Liaoning provinces is far fewer than that in regions where marathons are not suitable. 

## 5. Conclusions

Different natural environmental elements have different impacts on marathon events. This article uses GIS spatial information analysis to study the impact of natural environment on the spatial distribution of marathon events in China and the impact of each natural environmental element’s on events. The conclusions are: (1) In terms of terrain, low-lying areas are more favoured for marathon events. In China, the third terrain step is favoured for events over the second step, and the second step is more suitable than the first step. Among the five major terrains, plains are the most suitable for marathon running, followed by basins, hills, and mountains, while plateaus are not suitable for marathon running; (2) In terms of climatic conditions, subtropical monsoon climate and temperate monsoon climate regions are more suitable for marathon running. If the marathon running is to be held in temperate continental climate and tropical monsoon climate regions, the timing should be well considered. It’s better to choose a time when the temperature is between 14°C to 16°C, the humidity is between 30% to 60%, the air pressure is between 1015 to 1023 hPa, and the wind speed is between 2 to 5 m/s. It is not suggested to host marathon running in cities with the third or fourth tier air quality [43]. It is better to hold marathon running in the first and the second tier cities; (3) Currently, not many marathon running are held near water resources and mountains, but considering the purpose of urban marathon running in China, more marathon running should be held near water resources. Meanwhile, it is worth considering to hold the entertaining competitions such as trail racing and obstacle racing near mountain resources and thus more participants would be attracted by natural environmental resources. 

## 6. Limitations and Future Research

The purpose of this study is to explore cities suitable for holding marathon events from the perspective of natural environment, and to obtain indexes or conditions for reference, so as to provide references for cities that are willing to hold marathon events or some aspects for improvement for cities that are already holding marathon events. But there are still some limitations, first of all, owing to numerous natural environmental elements and the length of the article is limited, it is not possible to analyze each natural environment element’s impact on marathon running. Second, the terrain, topography and climate in the study are Categorical (nominal), In ArcGIS mapping, explanatory variables are not set in the map data and a unique ID field is uniformly used, so spatial regression modeling cannot be done. In addition, the geographical location of the marathon in the study is the point element, while the actual marathon course should be the line element, which may lead to errors in the results. Finally, this study only considered the relationship between natural environmental resources and the distribution of marathon events, and did not consider the human environment.

After the Chinese government streamlined the administration of sports events, cities no longer need national approval to host marathons, and more cities have the freedom of choice to host marathons [62]. But after those lousy marathons, more cities may need to hold back. Marathon will not be held overnight, but rather a professional thing, which requires unremitting input and professional operation. Even in other cities with 20 or 30 years of experience in marathon organization, the input to the race is a continuous process. Many Chinese cities, on the other hand, expect much more from marathon revenue than from daily event preparation and operation. Therefore, in the future, research can first explore the specific indicators or evaluation of marathon events held in Chinese cities from the perspective of public health. Future research can also explore the impact of China’s cultural environment on the holding of marathon events. From 2010 to 2018, the spatial distribution of marathon events in China can be found that, in addition to natural environmental resources, the cultural environment (population, policy, economy) also has a great impact on the holding of marathon events [62]. At the same time, the research ideas can also be applied to other countries, or even the world’s famous marathon distribution research, and extend the findings from the present study.

## Figures and Tables

**Table 1:** Marathon events in China in 2018 (by month).

**Month**	**January**	**February**	**March**	**April**	**May**	**June**
Number of events	4	0	22	47	39	18
Participants	60,000	0	303,500	408,100	328,000	129,640
**Month**	**July**	**August**	**September**	**October**	**November**	**December**
Number of events	12	12	27	60	66	35
Participants	80,000	112,000	165,150	593,500	762,800	419,900
		**Events Hosted in each Province**				
Jiangsu (40), Shandong (27), Zhejiang (23), Guangdong (22), Hubei (21), Anhui (20), Sichuan (18), Henan (15), Yunnan (15), Beijing (12), Hebei (11), Jiangxi (11), Shaanxi (11), Guangxi (10), Fujian (9), Chongqing (9), Shanghai (8), Gansu (8), Shanxi (7), Hainan (7), Inner Mongolia (7), Heilongjiang (6), Liaoning (5), Hunan (5), Guizhou (5), Jilin (5), Tianjin (2), Tibet (1), Ningxia (1), Xinjiang (1), Qinghai (0)						

**Table 2:** Main gathering area on each terrain step and the distribution of marathon events on different landforms.

**Terrain Step**	**Main Gathering Areas**		
1st	Marathon running are not gathered		
2nd	Sichuan Basin, part of Yunan-guizhou Plateau, near Hexi Corridor		
3rd	the Yangtze River Delta, the Yangtze plain, North China plain, Lingnan		
**Distribution of Marathon Running on Different Landforms**			
Landform	Number	Landform	Number
Plain	193	Hill	57
Plateau	37	Mountain	28
Basin	27		

**Table 3:** Distribution of marathon running in different climatic area and air quality levels.

**Climate**	**Number**	**Climate**	**Number**
Subtropical monsoon climate	205	Temperate monsoon climate	109
Temperate continental climate	19	Tropical monsoon climate	8
Plateau alpine climate	1		
**Marathon Running Distribution by Regional Air Quality**			
**Tier** **(** **Air quality index** **)**	**Province (** **Amount)**		**Total Number (%)**
1st (0–29)	Hainan (7), Guizhou (5), Yunnan (15), Tibet (1), Fujian (9), Heilongjiang (6)		43 (12.5%)
2nd (30–39)	Guangdong (22), Shanghai (8), Guangxi (10), Hunan (5), Jiangxi (11), Zhejiang (23), Sichuan (18), Qinghai (0), Gansu (8), Inner Mongolia (7), Jilin (5)		117 (34.2%)
3rd (40–49)	Chongqing (9), Hubei (21), Anhui (20), Jiangsu (40), Ningxia (1), Shaanxi (11), Liaoning (5), Beijing (12)		119 (34.8%)
4th (50–56)	Hebei (11), Henan (15), Shanxi (7), Shandong (27), Tianjin (2), Xinjiang (1)		63 (18.5%)

**Table 4:** Intersect output of the buffer radius of water resources/mountains and marathon running.

**Buffer Radius of Main Rivers**	**Buffer Radius of Main Lakes**	**Total Intersection**	**%**
500 m	1000 m	24	7.0%
3 km	5 km	131	38.3%
5 km	10 km	191	55.8%
10 km	20 km	298	87.1%
**Buffer Radius of Main Mountains**		**Total Intersection**	**%**
20 km		13	3.8%
50 km		39	11.4%

**Table 5:** Regression analysis.

	R	R^2^	β	*p*
Buffer radius of main rivers	0.984	0.968	0.028	0.016
Buffer radius of main lakes	0.977	0.954	0.014	0.023
Buffer radius of main mountains	1.000	1.000	0.867	0.000

**β**: Bate is the standardized partial regression coefficient.
